# BandHiC: a memory-efficient and user-friendly Python package for organizing and analyzing Hi-C matrices down to sub-kilobase resolution

**DOI:** 10.1186/s12864-026-12680-4

**Published:** 2026-05-06

**Authors:** Weibing Wang, Junping Li, Yusen Ye, Lin Gao

**Affiliations:** Department of Computer Science, School of Computer Science and Technology, Xidian University, Xi’an, Shaanxi China

**Keywords:** 3D genome, Hi-C, Software, Python package, Data structure

## Abstract

**Background:**

Recent advances in high-resolution Hi-C and Micro-C technologies have enabled finer-scale characterization of 3D genome architecture. However, these improvements also introduce substantial computational challenges, as the memory requirements of Hi-C/Micro-C contact matrices scale quadratically with resolution, leading to prohibitive resource consumption.

**Results:**

To address this, we developed BandHiC, a memory-efficient and user-friendly Python package for organizing and analyzing Hi-C matrices down to sub-kilobase resolution. BandHiC adopts a banded storage strategy that preserves only a configurable diagonal bandwidth of the dense contact matrix, reducing memory usage by up to 99% while maintaining fast random access and intuitive indexing operations. In addition, it provides flexible masking mechanisms to handle missing values, outliers, and unmappable regions, and supports efficient vectorized operations optimized with NumPy, thereby enabling scalable analysis of ultra-high-resolution Hi-C datasets.

**Conclusions:**

BandHiC provides a memory-efficient and scalable framework that enables sub-kilobase-resolution Hi-C matrix analysis on standard hardware. Its seamless integration with the NumPy ecosystem and user-friendly design make it a practical and accessible foundation for future advances in 3D genomics. The source code of the BandHiC Python package is publicly available on GitHub (https://github.com/xdwwb/BandHiC-Master*)*, and comprehensive documentation is provided at its website (https://xdwwb.github.io/BandHiC-Master/*).* Installation can be performed conveniently through Python’s pip package manager.

**Supplementary Information:**

The online version contains supplementary material available at 10.1186/s12864-026-12680-4.

## Background

High-throughput chromosome conformation capture (Hi-C) [1–4] and its variants, such as Micro-C [5–7], have substantially advanced our understanding of genome architecture by enabling genome-wide mapping of chromatin interactions at progressively higher resolutions. Hi-C data typically measures the interaction frequency between evenly spaced chromatin segments, represented mathematically as a contact matrix, where each bin corresponds to a genomic interval and the bin size defines the resolution of the contact map. Over the past decade, improvements in sequencing throughput and experimental protocols have enabled a dramatic increase in Hi-C data resolution, from early megabase (Mb)-scale maps to kilobase (kb)- and even sub-kilobase (e.g., 500 bp, 250 bp) contact matrices [4, 6]. More recently, single-cell Micro-C has achieved contact maps at resolutions as high as 5 kb [8]. These advances in resolution have facilitated the discovery of finer-scale chromatin structures and their regulatory roles in gene expression [3, 6, 7, 9, 10].

However, higher-resolution data generated by these assays pose significant computational challenges, particularly in terms of memory consumption [11, 12]. For instance, loading a dense Hi-C matrix into Random Access Memory (RAM) at 1 kb resolution for the human genome (~ 3 billion base pairs) would require approximately $\:{\left(3\times\:{10}^{9}/{10}^{3}\right)}^{2}\times\:8\:\mathrm{b}\mathrm{y}\mathrm{t}\mathrm{e}\mathrm{s}=7.2\times\:{10}^{13}\:\mathrm{b}\mathrm{y}\mathrm{t}\mathrm{e}\mathrm{s}=72\:\mathrm{t}\mathrm{e}\mathrm{r}a\mathrm{b}\mathrm{y}\mathrm{t}\mathrm{e}\mathrm{s}$ of memory ($\:\approx\:$ 66 tebibytes), assuming double-precision floating-point representation (8 bytes per entry). Even when restricting the analysis to the longest human chromosome (chromosome 1, ~ 249 Mb), a dense matrix at 1 kb resolution would still require nearly 462 GiB of memory. In practical workflows, intermediate data structures generated during normalization and downstream analyses often lead to a significant increase in peak memory usage beyond the raw matrix itself. Such memory demands far exceed the capacities of most computational environments. This issue becomes even more pronounced at sub-kilobase or single-cell resolutions, rendering dense matrix representations impractical for many real-world applications. These limitations highlight the need for more memory-efficient data structures tailored for high-resolution Hi-C data.

Many current methods for identifying genome structural patterns, such as chromatin loops and topologically associating domains (TADs), rely heavily on dense matrix representations to facilitate rapid, random data access during computation [13]. Tools, such as TopDom [14], MSTD [15], DeTOKI [16] and SnapHiC [17], exhibit high memory consumption associated with dense matrices, which limits their scalability to higher-resolution Hi-C datasets. Although conventional sparse matrix formats can reduce memory usage, they lack efficient random-access capability, significantly slowing downstream analyses and complicating algorithm design, particularly for tasks requiring frequent element-wise access or submatrix extraction. Other methods, including Mustache [18] and Chromosight [19], attempt to circumvent this limitation by partitioning the full dense matrix into smaller blocks that are loaded into memory on demand. However, these methods introduce added implementation complexity and require careful memory management to maintain computational efficiency.

However, these methods typically leverage high-resolution Hi-C data predominantly within short-range genomic distances (typically within 10 Mb), as chromatin loops and TADs are generally constrained to such scales [3]. For example, TopDom detects TADs by computing an insulation-like signal within a near-diagonal sliding window, which targets domain-length scales on the order of a few hundred kilobases [14]. HiCCUPS reports that 98% of detected loops occur between loci < 2 Mb apart; accordingly, its CPU implementation adopts an 8 Mb default diagonal bandwidth as a practical compromise to reduce computation time while covering most candidate loop distances [3]. Consistently, Arrowhead identifies contact domains spanning 40 kb–3 Mb (median 185 kb) [3]. Other widely used approaches also operate under bounded distances: Fit-Hi-C focuses on interactions at an intermediate scale of ~ 50 kb–10 Mb [20], Peakachu (a machine-learning–based loop caller) uses a default upper genomic distance cutoff of a few megabases [21], and chromatin loop or Hi-C map prediction methods often restrict candidate pairs to ~ 1–2 Mb [22–25]. Together, these practices motivate focusing computation on band-limited regions for high-resolution cis analyses. However, efficient data-structure support for such workflows at sub-kilobase resolution remains limited, motivating the design of BandHiC.

Interactions at distances beyond a few megabases become increasingly sparse at high resolutions and are often irrelevant to local structures such as loops and TADs. Thus, focusing on short-range contacts is both computationally and biologically justified. Therefore, there is an urgent need for a novel memory scheme tailored specifically for local-range higher-resolution Hi-C data, one that dramatically reduces memory usage while retaining the fast random-access capabilities of dense matrices.

Built upon NumPy [26, 27], a fundamental library for numerical computing in Python, we developed BandHiC, a memory-efficient Python package specifically designed for organizing and analyzing short-range contacts of Hi-C data down to sub-kilobase resolutions. BandHiC adopts a banded matrix storage scheme that stores only a configurable diagonal bandwidth of the full Hi-C contact matrix. A banded matrix is a standard concept in numerical analysis, referring to matrices whose nonzero entries are confined near the main diagonal [28], and scientific computing libraries such as SciPy provide linear system solvers that operate on banded storage formats [29]. BandHiC preserves efficient random-access capabilities by employing a direct index mapping between the banded and dense matrix representations, while supporting familiar NumPy-style indexing semantics (slicing, Boolean array indexing, integer array indexing) to facilitate user-friendly and efficient data access. BandHiC also integrates masking functionality akin to NumPy’s MaskedArray module, enabling straightforward handling of gaps, outliers, and other aberrant values in Hi-C matrices. Finally, BandHiC supports diverse numerical operations optimized through NumPy’s efficient vectorized computations, thus offering both memory efficiency and high computational performance essential for practical high-resolution Hi-C data analysis.

## Implementation

### Data representation

**Fig. 1:**
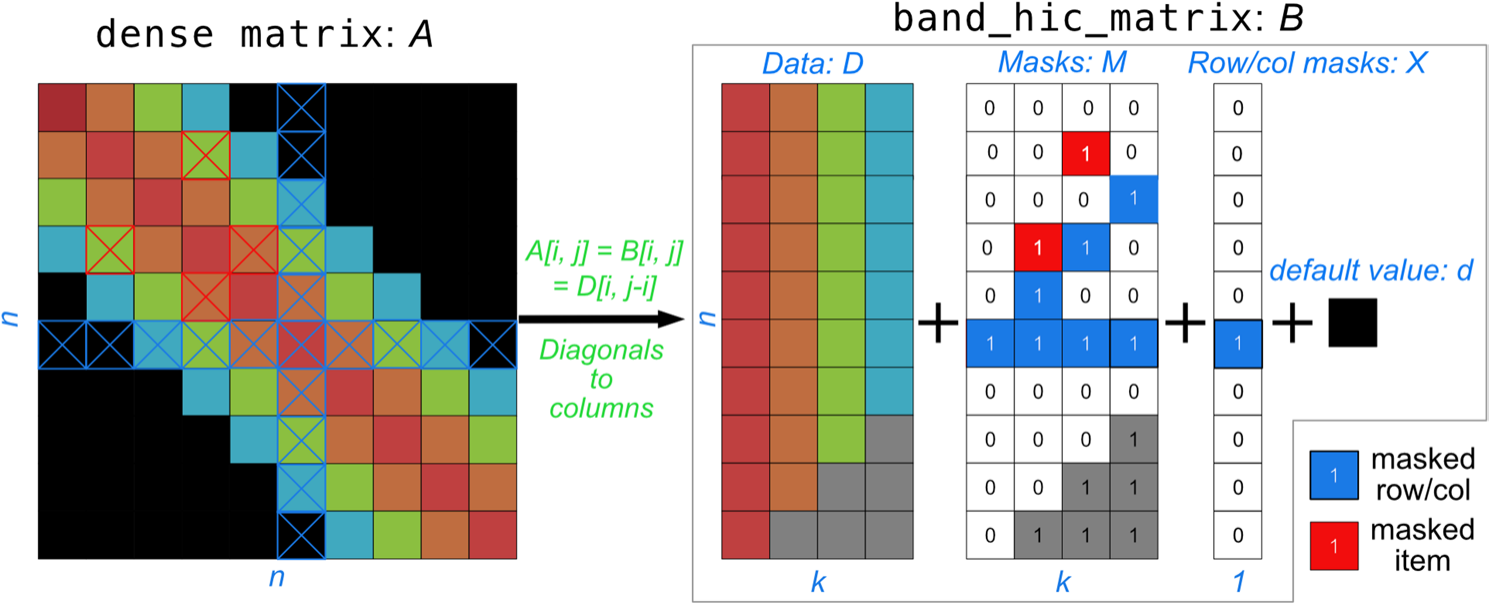
Data model of BandHiC. Schematic illustration of converting a dense symmetric matrix *A* into a banded representation consisting of a data matrix *D*, an element-wise mask matrix *M*, a row/column mask matrix *X*, and a default value *d* for out-of-band entries. Diagonal elements from *A* are reorganized into columns of *D*; *M* marks missing or outlier entries; *X* indicates masked rows or columns

To address the increasing memory demands posed by high-resolution Hi-C data, we introduce band_hic_matrix, the core class implemented in the BandHiC package. Given a Hi-C contact matrix $\:A\in\:{R}^{n\times\:n}$ at resolution $\:r$, band_hic_matrix retains only the diagonals within a user-defined bandwidth $\:k$, yielding a compact representation $\:D\in\:{R}^{n\times\:k}$ (Fig. 1). This format ensures that each column in $\:D$ corresponds to a fixed diagonal of $\:A$, such that the mapping$\:|i-j|<k$.

The memory efficiency achieved by this strategy is substantial. When $\:k\ll\:n$, the memory footprint of band_hic_matrix is reduced from $\:\mathcal{O}\left({n}^{2}\right)$ to $\:\mathcal{O}\left(nk\right)$. For example, assuming a resolution of 1 kb and a bandwidth of 2 Mb ($\:k=2000$), the representation of chromosome 1 of the human genome (~ 249 Mb) requires 3.7 GiB of memory, less than 1% of the memory required by the dense matrix (~ 462 GiB). This compression makes high-resolution Hi-C data accessible even on commodity hardware, without compromising the efficiency of random data access.

To further enhance the flexibility of usage, band_hic_matrix supports an optional two-layer masking mechanism. An element-wise mask matrix $\:M\in\:\{\mathrm{0,1}{\}}^{n\times\:k}$ allows users to selectively ignore missing or outlier contacts, enabling robust statistical estimation on unmasked subsets. Additionally, a bin-level mask $\:X\in\:\{\mathrm{0,1}{\}}^{n}$ supports the exclusion of entire rows or columns, particularly useful for removing repetitive genomic regions lacking valid Hi-C signals. These masking features facilitate downstream tasks such as estimation of average contact intensity at specific genomic distances, while preserving statistical validity.

Lastly, a scalar default value $\:d$ is defined to fill in the undefined entries of $\:A$ not covered by the banded matrix $\:D$. This default is typically set to 0, consistent with the assumption that long-range interactions are negligibly sparse. The advantage of using default values is that, in addition to treating the band_hic_matrix as a full dense matrix for indexing and conversion with a dense matrix, it also allows out-of-band entries to participate in mathematical operations. For example, when adding 1 to the band_hic_matrix object, not only are the in-band entries incremented, but the default value representing the out-of-band entries also increases by 1. Together, the components $\:D$, $\:M$, $\:X$, and $\:d$ allow for seamless reconstruction of the dense matrix $\:A$ when required. Overall, band_hic_matrix provides an efficient, flexible data representation for scalable Hi-C data analysis.

### BandHiC package

**Fig. 2:**
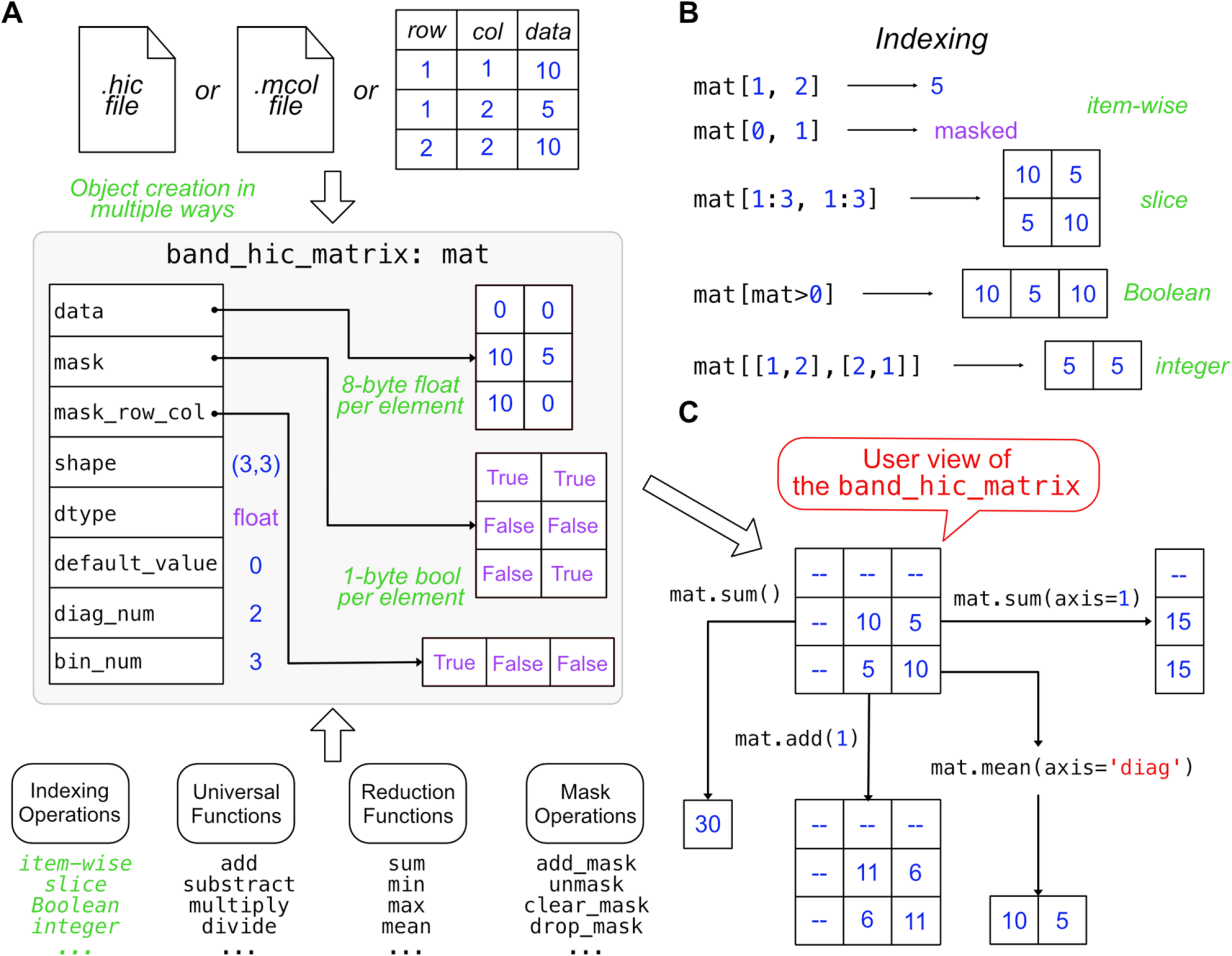
Overview of the BandHiC package. **A** Example of a band_hic_matrix object in the BandHiC package. **B** Indexing methods supported by BandHiC. **C** Example of computation methods supported by BandHiC

BandHiC is distributed as an open-source Python package under the MIT license. It is compatible with Python version 3.8 or higher and can be deployed on Linux and macOS platforms. The BandHiC package relies primarily on NumPy and SciPy, which provide the computational backbone for the banded matrix data structure and its core operations. In addition, BandHiC wraps the file-reading functions of cooler and hic-straw, allowing it to directly read .hic, .cool, or .mcool files as inputs for creating a band_hic_matrix object (Fig. 2A).

BandHiC primarily defines a matrix class, band_hic_matrix, which represents Hi-C contact data in a banded matrix representation. Each instance contains a numerical NumPy array of shape (bin_num, diag_num), together with Boolean arrays mask and mask_row_col that record element-wise and row/column-wise exclusions. Regarding the choice of diag_num, based on common Hi-C analysis workflows [3, 14], we recommend setting it to a diagonal bandwidth corresponding to ~ 2–8 Mb, depending on the resolution. In practice, 2 Mb is typically sufficient for TAD calling, whereas 2–8 Mb is recommended for loop calling. Users can also flexibly adjust this parameter according to their specific tasks and objectives.

Although the mask array has the same shape as the data array, it consumes significantly less memory. Specifically, the data array is typically stored using double-precision floating-point or int64 values (8 bytes per entry), whereas the mask array only requires a Boolean representation (1 byte per entry). As a result, the memory footprint of the mask array is only one eighth that of the data array. In contrast, the mask_row_col array is a one-dimensional vector, whose memory consumption is negligible compared with that of the data array. Elements outside the stored bandwidth are represented by a scalar default_value, allowing the matrix to behave as a dense symmetric array while avoiding redundant storage. Objects can be constructed from .hic, .mcool files, or from triplet-form contact records (rows, columns, and contact frequencies) (Fig. 2A).

The package fully supports NumPy-style indexing (Fig. 2B), including item-wise, slice, Boolean array, and integer array indexing. It also provides a series of methods and functions for constructing, manipulating, and performing computations on the band_hic_matrix objects (Fig. 2C). The design and implementation of the indexing and computational operations supported by BandHiC are described in detail in the following two subsections.

### Indexing operations

Building on the direct coordinate mapping between the banded representation D and the full dense matrix A described above (Fig. 1), band_hic_matrix provides constant-time random access to all in-band entries while preserving dense-matrix semantics. In practice, users can access elements through $\:B[i,\:j]=D[i,\:j-i]=A[i,\:j]$, and interact with the object as if it were a standard NumPy array, without being exposed to the underlying storage scheme.

Leveraging this property, band_hic_matrix supports full NumPy-style indexing operations, including slicing, Boolean array, and integer array indexing (Fig. 2B). Slicing selects contiguous ranges of data, such as $\:A[2:5]$. Boolean array indexing extracts elements that meet specific conditions, for example $\:A[A>0]$. Integer array indexing allows arbitrary selection using arrays of integer indices, such as $\:A\left[\right[0,\:2,\:4\left]\right]$. This design allows users to easily query local chromatin contacts and provides a flexible and efficient framework for data manipulation in scientific computing. For instance, a slice operation such as $\:B[i:j,\:i:j]$ or $\:B[i:j]$ retrieves a banded submatrix. Combined with the todense operation, this enables reconstruction of the dense submatrix for downstream analysis or visualization.

For a band_hic_matrix object with the mask array, indexing operations return either a ma.MaskedArray object or the masked constant. In NumPy, a MaskedArray stores numerical data together with a Boolean mask that marks missing or invalid entries. In practice, indexing a band_hic_matrix behaves as if operating directly on a MaskedArray, thereby providing users with considerable flexibility.

Indexing operations that fall outside the predefined diagonal bandwidth do not raise errors; instead, such entries are filled with a user-specified default value (e.g., zero), further improving robustness and usability in practical applications.

### Numerical computation

In addition to flexible data access, band_hic_matrix also supports a wide range of numerical operations, including element-wise mathematical operations and reduction operations (Fig. 2C). BandHiC is built on top of NumPy, which provides the foundation for efficient numerical computation in Python. NumPy offers high-performance, C-optimized universal functions (ufuncs) that perform element-wise operations with support for broadcasting and type casting. By leveraging these ufuncs, BandHiC implements 71 element-wise mathematical operations directly on the band_hic_matrix (Table 1). This design eliminates the need for explicit Python loops, ensures full compatibility with the NumPy ecosystem, and significantly improves computational speed. Consequently, BandHiC inherits the scalability and efficiency of NumPy, enabling fast and memory-efficient analysis of large genomic contact matrices. Moreover, NumPy provides interfaces for defining custom array-like objects while maintaining seamless integration with NumPy, enabling BandHiC to implement specialized matrix types efficiently and flexibly. By building on NumPy in this way, BandHiC inherits both its computational efficiency and its flexible programming model, making it well-suited for scalable analysis of large-scale Hi-C data.

**Table 1:** Universal functions that BandHiC supports

Function	Description	Function	Description
absolute	Absolute value	add	Element-wise addition
arccos	Inverse cosine	arccosh	Inverse hyperbolic cosine
arcsin	Inverse sine	arcsinh	Inverse hyperbolic sine
arctan	Inverse tangent	arctan2	Arctangent of y/x with quadrant
arctanh	Inverse hyperbolic tangent	bitwise_and	Element-wise bitwise AND
bitwise_or	Element-wise bitwise OR	bitwise_xor	Element-wise bitwise XOR
cbrt	Cube root	conj	Complex conjugate
conjugate	Alias for conj	cos	Cosine function
cosh	Hyperbolic cosine	deg2rad	Degrees to radians
degrees	Radians to degrees	divide	Element-wise division
divmod	Quotient and remainder	equal	Element-wise equality test
exp	Exponential	exp2	Base-2 exponential
expm1	exp(x) − 1	fabs	Absolute value (float)
float_power	Floating-point power	floor_divide	Integer division (floor)
fmod	Modulo operation	gcd	Greatest common divisor
greater	Element-wise greater-than test	greater_equal	Greater-than or equal test
heaviside	Heaviside step function	hypot	Euclidean norm
invert	Bitwise inversion	lcm	Least common multiple
left_shift	Bitwise left shift	less	Element-wise less-than test
less_equal	Less-than or equal test	log	Natural logarithm
log1p	log(1 + x)	log2	Base-2 logarithm
log10	Base-10 logarithm	logaddexp	log(exp(x) + exp(y))
logaddexp2	Base-2 version of logaddexp	logical_and	Element-wise logical AND
logical_or	Element-wise logical OR	logical_xor	Element-wise logical XOR
maximum	Element-wise maximum	minimum	Element-wise minimum
mod	Remainder (modulo)	multiply	Element-wise multiplication
negative	Element-wise negation	not_equal	Element-wise inequality test
positive	Returns input unchanged	power	Raise to power
rad2deg	Radians to degrees	radians	Degrees to radians
reciprocal	Element-wise reciprocal	remainder	Modulo remainder
right_shift	Bitwise right shift	rint	Round to the nearest integer
sign	Sign of input	sin	Sine function
sinh	Hyperbolic sine	sqrt	Square root
square	Square of input	subtract	Element-wise subtraction
tan	Tangent function	tanh	Hyperbolic tangent
true_divide	Division that returns a float		

Reduction operations refer to functions that aggregate multiple values into a single result. They can be applied globally to all elements of a matrix, or along specific axes to summarize rows or columns. Examples include sum, min, max, and mean. BandHiC supports ten such reduction operations (Table 2), which work along conventional axes (rows or columns) in the same way as NumPy. In addition, BandHiC extends these operations to the diagonal axis—a feature not available in NumPy. This diagonal reduction is particularly useful for Hi-C data, as it allows interaction frequencies to be summarized by genomic distance, thereby supporting distance-dependent normalization and analyses such as distance-decay profiling. All operations remain fully compatible with masked band_hic_matrix objects, ensuring robust handling of missing or low-quality data in large-scale Hi-C analysis.

The implementation of reduction operations in BandHiC is designed to behave equivalently to those on a dense matrix, but without explicitly constructing the dense matrix, which would otherwise consume substantial memory. During computation, BandHiC automatically fills out-of-band entries with the default value, symmetrizes interactions in the lower-triangular part of the matrix, and excludes entries masked by either element-wise or row/column masks.

**Table 2:** Reduction functions that BandHiC supports

Function	Description
sum	Compute the sum of all elements along the specified axis
prod	Compute the product of all elements along the specified axis
min	Return the minimum value along the specified axis
max	Return the maximum value along the specified axis
mean	Compute the arithmetic mean along the specified axis
var	Compute the variance (average squared deviation)
std	Compute the standard deviation (square root of variance)
ptp	Compute the range (max - min) of values along the axis
all	Return True if all elements evaluate to True
any	Return True if any element evaluates to True

Taken together, band_hic_matrix combines the memory efficiency of a banded storage model with the expressiveness of NumPy’s interface. By mimicking both Numpy’s ndarray and MaskedArray behaviors, it provides an intuitive and powerful interface for users, substantially lowering the barrier to adoption and enabling seamless integration into existing Hi-C data analysis pipelines. Please refer to BandHiC’s website for a detailed list of all supported functions and tutorials.

## Results

### Performance benchmarking

To verify correctness, we compared BandHiC outputs with NumPy arrays using an automated test suite covering core numerical operations as well as indexing and masking behaviors. Across all tests, BandHiC produces results identical to NumPy or consistent within numerical precision, ensuring user-level output equivalence. All test scripts are publicly available in the project’s GitHub repository.

**Fig. 3:**
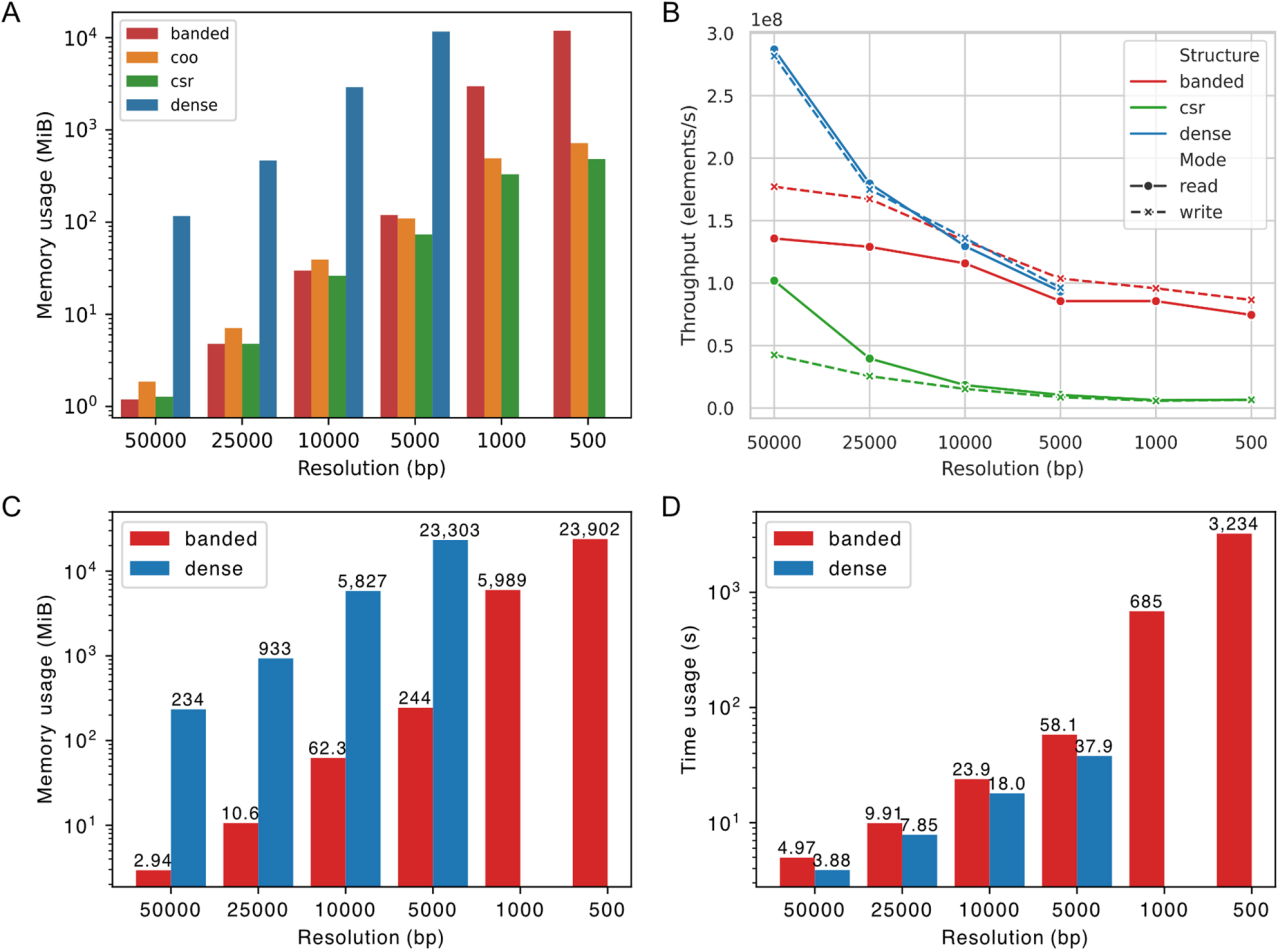
Evaluation of BandHiC. **A** Memory usage comparison of different matrix representations, including banded, dense, COO (coordinate format), and CSR (compressed sparse row), on mouse embryonic stem cell (mESC) Micro-C data (chromosome 1) at various resolutions. **B** Read and write throughput comparison between banded, CSR, and dense matrix representations on mESC Micro-C data (chromosome 1). Throughput is measured as the number of matrix elements accessed per second (elements/s). COO is not included because it does not support efficient access to individual matrix entries required for this benchmark. **C** Memory usage comparison between the banded and dense matrices when running TopDom on mESC Micro-C data (chromosome 1). **D** Runtime comparison for the same task. At 1,000 bp and 500 bp resolution, the dense representations failed due to memory overflow

To evaluate the efficiency of BandHiC for random, element-wise access to Hi-C contact matrices, we compared its memory footprint and read/write throughput with three commonly used representations: dense array, CSR (compressed sparse row), and COO (coordinate format), across resolutions from 50 kb to 500 bp on mouse embryonic stem cell (mESC) Micro-C data from chromosome 1 (Fig. 3A, B). For a fair comparison, both CSR and COO were restricted to store only interactions within the same diagonal bandwidth as BandHiC, and all random-access benchmarks were limited to entries inside this band (see Supplementary Material 2 for detailed experimental setup). We benchmarked element-wise random access on in-memory Hi-C matrices, reflecting the fine-grained local access pattern commonly used in Hi-C local interaction analysis (e.g., loop and TAD detection). Under other access patterns or operations (e.g., row-wise iteration or matrix operations), CSR and COO may show different relative performance.

Dense matrices serve as an ideal baseline for access performance, as their contiguous memory layout enables highly efficient traversal. When feasible, dense representations indeed achieve the highest read and write throughput, except at 5 kb resolution, where their write throughput is surpassed by the banded representation. However, their memory footprint grows quadratically with resolution, making them impractical at high resolutions: in our experiments, dense matrices could not be instantiated at 1 kb and 500 bp due to memory exhaustion. In contrast, across the tested resolutions, BandHiC achieves up to ~ 99% reduction in memory usage relative to dense representations, while retaining approximately 47–108% of the dense read/write throughput (Fig. 3A, B), providing a scalable alternative for high-resolution analyses.

The CSR representation substantially reduces memory usage by storing only nonzero entries together with row pointers, but this comes at the cost of access efficiency. Because retrieving an individual element requires pointer indirection and index searches within each row, CSR exhibits consistently lower element-wise throughput. Across all tested resolutions, BandHiC achieves approximately 1.3–13.5× higher read throughput and 4.2–16.6× higher write throughput than CSR under the same band-limited setting. For example, at 1 kb resolution, BandHiC shows approximately 13× higher read throughput and 17× higher write throughput than CSR. These results indicate that CSR performance degrades sharply under highly sparse conditions, whereas BandHiC maintains stable and high access rates.

In terms of memory usage, both BandHiC and CSR dramatically reduce storage requirements relative to dense matrices, but their relative efficiency depends on the density of interactions within the band. At coarse resolutions (e.g., 50 kb and 25 kb), where the banded region is relatively dense, BandHiC can be even more memory-efficient than CSR because it stores only the banded values without any pointer or index overhead. For example, at 50 kb resolution BandHiC requires ~ 1.19 MiB, slightly less than CSR (~ 1.27 MiB), and at 25 kb both formats use comparable memory (~ 4.77 vs. ~4.79 MiB). As the resolution increases and the banded region becomes sparser, CSR becomes more memory-efficient than BandHiC (e.g., ~ 73.4 MiB vs. ~119.2 MiB at 5 kb), reflecting the growing advantage of skipping zero entries in sparse storage.

The COO representation offers an even simpler sparse storage scheme by explicitly listing the coordinates and values of nonzero entries, resulting in low memory overhead and flexibility for constructing sparse matrices. However, it lacks an efficient interface for direct single-element access, as retrieving an entry would require scanning coordinate lists. Consequently, COO is not included in the throughput benchmark, as it is not compatible with the element-wise access pattern evaluated here. This reflects a fundamental trade-off: while COO is advantageous for matrix assembly and storage, it is ill-suited for random-access workloads in downstream Hi-C analysis.

We repeated the same benchmarking on GM12878 Hi-C data [3], and obtained consistent results that further confirm the advantages of BandHiC (Supplementary Fig. S1A, B). Overall, these results demonstrate that BandHiC provides a favorable compromise between dense and sparse representations. It dramatically improves scalability over dense matrices by reducing memory requirements by up to two orders of magnitude, while preserving a large fraction of dense access performance, and it substantially outperforms CSR in element-wise throughput under the same storage constraints. By combining compact banded storage with direct coordinate mapping and $\:\mathcal{O}\left(1\right)$ access semantics, BandHiC enables efficient random access to high-resolution Hi-C data at scales where existing formats become impractical.

### Application: reducing TopDom memory consumption

While the previous section demonstrates the core functionality and syntax of BandHiC, here we evaluate its practical utility by integrating it with the TAD-calling algorithm TopDom [14]. TopDom is an efficient and robust algorithm for detecting topologically associating domains (TADs). It uses a sliding window approach on Hi-C contact matrices to identify local minima in contact intensity, which mark potential TAD boundaries. Owing to its robustness and reproducibility, TopDom was rated as the best-performing TAD detection method in a benchmark study [9]; however, its reliance on dense matrices makes it difficult to apply to sub-kilobase resolution Hi-C data. The original TopDom algorithm was developed in the R language [14]. In BandHiC, we provide a Python implementation of TopDom as a built-in function, ensuring functional consistency with the original method while extending support for both NumPy’s ndarray and BandHiC’s band_hic_matrix class. This integration not only facilitates direct identification of TADs from high-resolution Hi-C data within the BandHiC but also enables a fair comparison of memory usage and runtime performance between dense and banded representations.

To evaluate the effectiveness of BandHiC, we benchmarked the TopDom algorithm on chromosome 1 of mouse embryonic stem cell (mESC) Micro-C data across multiple resolutions using both dense matrix and band_hic_matrix, with the band_hic_matrix bandwidth set to 2 Mb (see Supplementary Material 2 for detailed experimental setup). As shown in Fig. 3C, BandHiC substantially reduces memory usage at all tested resolutions. At 1000 bp and 500 bp resolution, dense matrix representations could not be constructed within the available system memory (60,268 MiB RAM and 12,288 MiB swap space), and the program terminated with memory allocation errors. In contrast, the band_hic_matrix version of TopDom completes successfully, with memory usage of only 5,989 MiB and 23,902 MiB at 1000 bp and 500 bp resolution, respectively.

The banded matrix introduces a modest increase in runtime compared to the dense matrix (Fig. 3D). This overhead arises not from masking operations (which were disabled for this evaluation) but from index computation performed during element access within the band_hic_matrix object. Overall, in our benchmarks, BandHiC uses approximately 1% of the memory of dense matrices, while incurring approximately a 50% increase in runtime compared with the dense implementation. Because TopDom’s default sliding window (~ 200 kb) is fully contained within the 2 Mb bandwidth used in BandHiC, the BandHiC-based and dense implementations yield identical TAD calls; therefore, we do not further assess TopDom’s detection accuracy here and instead focus on benchmarking memory usage and runtime efficiency. We repeated the same benchmarking on GM12878 Hi-C data, and obtained consistent results that further confirm the advantages of BandHiC (Supplementary Fig. S1C, D).

These results indicate that band_hic_matrix enables TAD-calling algorithms like TopDom to process high-resolution Hi-C data on a standard personal computer, making large-scale analysis feasible without incurring high computational cost. Owing to BandHiC’s NumPy-like API, other Hi-C-based pattern identification methods can be reimplemented with minimal modifications to the original code, thereby significantly improving their scalability and adaptability to higher-resolution Hi-C datasets.

### Usage examples

BandHiC can serve as an alternative to the NumPy package when managing and manipulating Hi-C matrices, aiming to address the issue of excessive memory usage caused by storing dense matrices with NumPy’s ndarray. At the same time, BandHiC supports masking operations like NumPy’s ma.MaskedArray module, with enhancements tailored for Hi-C data. Users can leverage their experience with NumPy when using the BandHiC package, so it is recommended that users have some basic knowledge of NumPy. Here are some code examples providing a quick guide and demonstration of the core functionalities of BandHiC:

**Figure:**
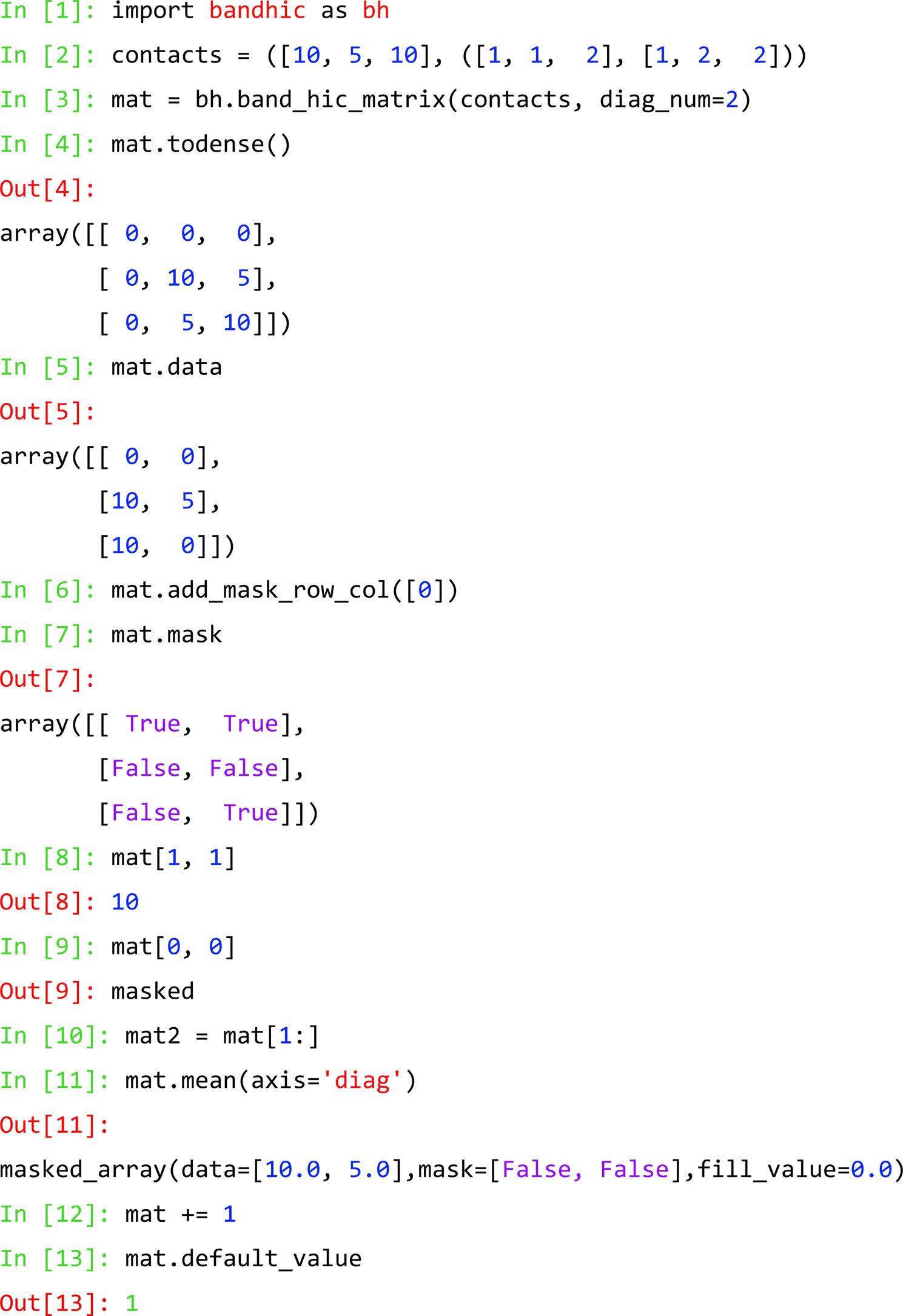

The defined band_hic_matrix object mat corresponds to the example shown in Fig. 2. The example is presented in the IPython-style interactive format, in which “In [n]:” indicates input commands and “Out[n]:” indicates the corresponding outputs.

## Conclusions

BandHiC alleviates the computational challenges of high-resolution Hi-C analysis through a banded storage scheme that reduces memory usage to ~ 1% of dense matrices while preserving constant-time random access. This enables domain-calling algorithms, such as TopDom, to run at sub-kilobase resolution on standard hardware. Seamless integration with the NumPy ecosystem, including support for universal functions, reductions, and Hi-C–specific diagonal operations, facilitates efficient distance-dependent analyses and easy adoption in existing pipelines. Furthermore, BandHiC provides a flexible programming model—supporting masking, user-defined default fill values, and robust handling of noisy or unmappable regions—which facilitates downstream analyses of local chromatin features such as loop and TAD detection.

BandHiC is designed for local interaction analysis in high-resolution Hi-C data, where most informative contacts fall within bounded genomic distances. By restricting storage to a user-defined interaction band, it achieves substantial memory reduction while preserving fast fine-grained random access and dense-matrix–like semantics, making it well suited for local-pattern analyses such as loop and TAD detection.

This band-limited design also defines its applicability boundaries. BandHiC is not intended for analyses that require full-matrix global interaction structures, such as A/B compartment, nor for normalization procedures such as KR normalization, which rely on whole-matrix constraints and extensive row-wise operations, where conventional sparse formats (e.g., CSR) are generally more appropriate. Overall, analyses of ultra long-range or inter-chromosomal interactions are inherently limited under the banded design, and future work may address this through hybrid banded–sparse storage strategies.

While BandHiC does not provide dedicated single-cell–specific analysis methods, single-cell Hi-C data can already be handled by loading each cell as an independent band_hic_matrix, with all supported operations identical to those used for bulk Hi-C. No specialized single-cell modeling or benchmarking is included in the current work. Looking forward, an explicit cell dimension extending the banded storage from two to three dimensions could offer more convenient and scalable support for large single-cell datasets and represents a possible direction for future development.

Taken together, BandHiC represents both a practical solution to the pressing computational challenges posed by ultra-high-resolution Hi-C data and a flexible foundation for future methodological advances. By combining memory efficiency, computational scalability, and a user-friendly interface, BandHiC has the potential to become a core component of the bioinformatics toolkit for 3D genomics.

## Availability and requirements

Project name

BandHiC.

Project home page: https://github.com/xdwwb/BandHiC-Master.

Operating system(s): Linux, macOS, and Windows (requires WSL).

Programming language: Python

Other requirements: Python 3.8 or higher

License: MIT

Any restrictions to use by non-academics: None

## Supplementary Information

Supplementary Material 1.

Supplementary Material 2.

## Data Availability

All the datasets used for this work are publicly available. The mESC Micro-C and GM12878 Hi-C data was downloaded from the Gene Expression Omnibus database (GEO; https://www.ncbi.nlm.nih.gov/geo/) under accession number GSE130275 and GSE63525.

## Abbreviations

Hi-C: High-throughput Chromosome Conformation Capture
Kb: Kilobase (1,000 base pairs)
Mb: Megabase (1,000,000 base pairs)
RAM: Random Access Memory
GiB: Gibibyte
TAD: Topologically Associating Domain
