# Supplementary material for: BandHiC: a memory-efficient and user-friendly Python package for organizing and analyzing Hi-C matrices down to sub-kilobase resolution
Source: BMC Genomics. 2026 May 6;27:567. doi: 10.1186/s12864-026-12680-4 (PMC13317261; doi:10.1186/s12864-026-12680-4)
Supplement: Supplementary file 1 — Supplementary Material 1. [file 12864_2026_12680_MOESM1_ESM.docx]

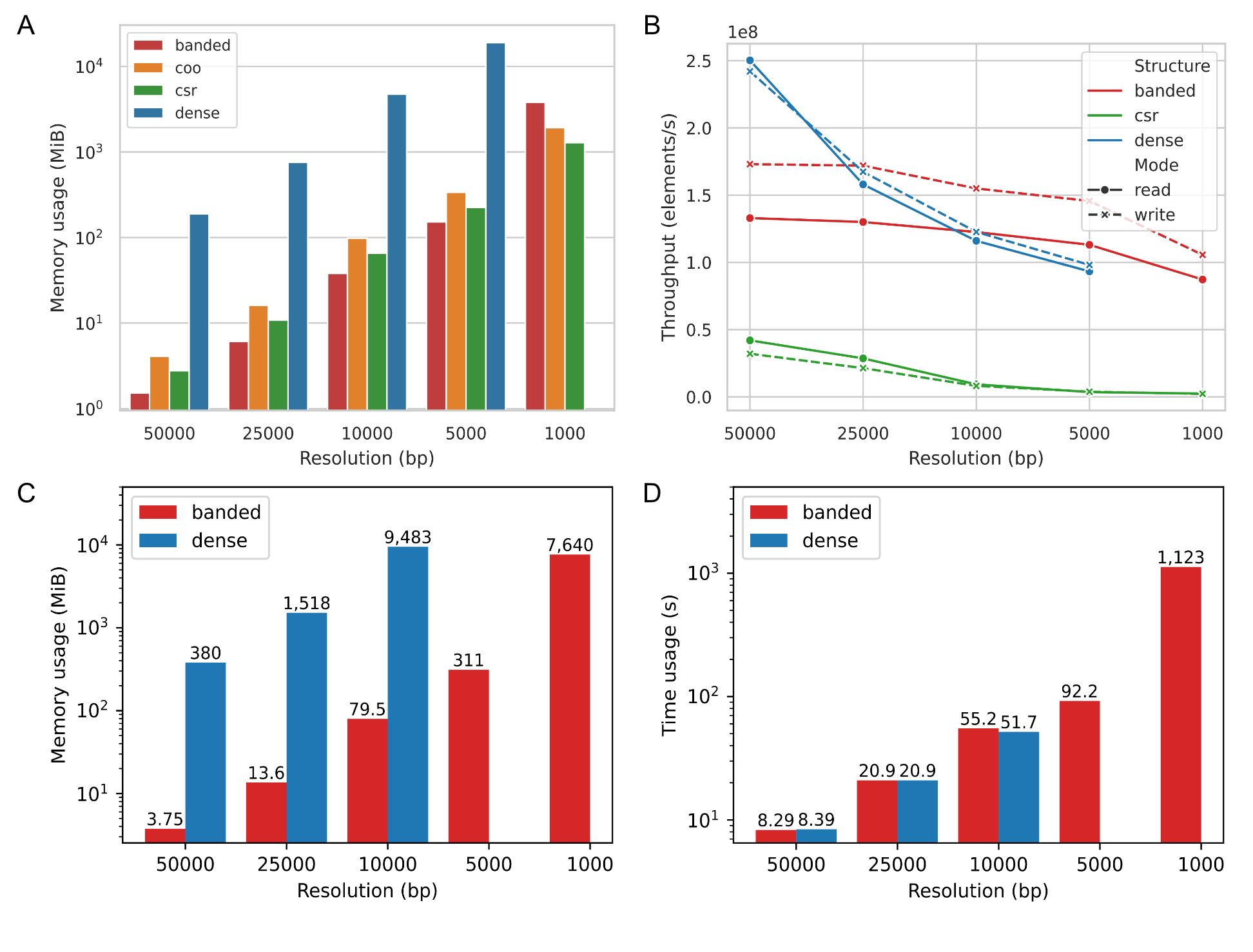


**Supplementary Figure S1. Evaluation of BandHiC.** (A) Memory usage comparison of different matrix representations, including banded, dense, COO (coordinate format), and CSR (compressed sparse row), on GM12878 Hi-C data (chromosome 1) at various resolutions. (B) Read and write throughput comparison between banded, CSR, and dense matrix representations on GM12878 Hi-C data (chromosome 1). Throughput is measured as the number of matrix elements accessed per second (elements/s). COO is not included because it does not support efficient access to individual matrix entries required for this benchmark. (C) Memory usage comparison between the banded and dense matrices when running TopDom on GM12878 Hi-C data (chromosome 1). (D) Runtime comparison for the same task. At 1,000 bp and 500 bp resolution, the dense representations failed due to memory overflow.
