# Supplementary material for: BandHiC: a memory-efficient and user-friendly Python package for organizing and analyzing Hi-C matrices down to sub-kilobase resolution
Source: BMC Genomics. 2026 May 6;27:567. doi: 10.1186/s12864-026-12680-4 (PMC13317261; doi:10.1186/s12864-026-12680-4)
Supplement: Supplementary file 2 — Supplementary Material 2. [file 12864_2026_12680_MOESM2_ESM.docx]

**Supplementary Material**

**Benchmark scripts, data availability, and computational environment**

**Benchmark scripts and execution**

All benchmark parameters are fully specified within the benchmark scripts. No command-line arguments are required. Each benchmark can be executed directly using:

python <path_to_benchmark_script>.py

Executing the script will automatically run all benchmark configurations evaluated in this study, including different data structures, access modes, and resolution settings. This design ensures that the benchmark procedure is self-contained and avoids inconsistencies arising from manual parameter specification.

For parameters that are not explicitly specified in the benchmark scripts, the default parameters provided by the corresponding modules in the BandHiC package are used. This guarantees consistency with standard BandHiC usage and ensures that the reported results reflect typical application scenarios.

The complete benchmark scripts are publicly available in the [BandHiC project repository](https://github.com/xdwwb/BandHiC-Master) under the benchmark/ directory.

**Data availability**

All benchmarks were performed using publicly available Hi-C data obtained from the Gene Expression Omnibus (GEO):

- **Dataset accession**: GSE130275 and GSE63525
- **File name**:
  - GSE130275_mESC_WT_combined_1.3B_microc.hic
  - GSE63525_GM12878_insitu_primary+replicate_combined.hic
- **URL**: https://www.ncbi.nlm.nih.gov/

The dataset was downloaded directly from GEO and processed according to the procedures described in the benchmark scripts. No additional proprietary or unpublished data were used.

**Software versions and dependencies**

All benchmarks were conducted using the following software environment:

- **Operating system**: Ubuntu 22.04 (WSL2)
- **Python**: Python 3.11
- **NumPy**: 1.26.4
- **SciPy**: 1.16.3
- **pandas**: 2.1.4
- **matplotlib**: 3.10.6
- **hic-straw:** 1.3.1
- **cooler:** 0.10.3
- **joblib:** 1.4.2
- **numba:** 0.61.2
- **BandHiC**: 0.3.0

All dependencies were installed from standard conda or pip channels. The BandHiC version corresponds to the implementation evaluated in this manuscript.

**Hardware configuration**

All experiments were performed on a single workstation with the following specifications:

- **CPU**: Intel Core i7-13700KF
- **Memory**: 64 GB DDR5
- **Memory speed**: 4000 MT/s
- **Architecture**: x86_64
- **Execution environment**: Linux (WSL2)
